# Night Shift Work and Vascular Function Parameters: A Systematic Review of Occupational and Healthcare Evidence with Implications for Radiographers

**DOI:** 10.12688/f1000research.182990.1

**Published:** 2026-06-30

**Authors:** Suneet Paulson, Suresh Sukumar, Poovitha Shruthi Paramshiva, Rajagopal Kadavigere, Winniecia Dkhar, Eswaran TPM, Nitika C. Panakkala, Abhimanyu Pradhan, Vaishali K, Basharan Chandrasekaran, Dilip Shettigara, Sathya Sabina Muthu, Koustubh Kamath, Sneha Ravichandran

**Affiliations:** 1 Department of Medical Imaging Technology, Manipal Academy of Higher Education, Manipal, Karnataka, 576104, India; 2 Division of Yoga, Manipal Academy of Higher Education, Manipal, Karnataka, 576104, India; 3 Department of Radio Diagnosis & Medical Imaging, Manipal Academy of Higher Education, Manipal, Karnataka, 576104, India; 4 Department of Physiotherapy, Manipal Academy of Higher Education, Manipal, Karnataka, 576104, India; 5 Department of Exercise and Sports Sciences, Symbiosis International University, Pune, Maharashtra, 412115, India

**Keywords:** Night shift work; Radiographers; Vascular function; Carotid intima-media thickness; Arterial stiffness; Circadian disruption; Endothelial function; Occupational health

## Abstract

**Background:**

Shift workers face elevated cardiovascular disease risk. Radiographers represent a unique occupational group exposed to both low-level ionizing radiation and chronic night shift work — each independently associated with subclinical vascular disease — yet no synthesis exists on vascular function in this population.

**Objective:**

To systematically identify and narratively synthesize evidence on associations between night shift work and objective vascular parameters — including CIMT, PWV, augmentation index, reactive hyperaemia index, and cerebrovascular white matter integrity — and assess applicability to radiographers.

**Methods:**

A systematic search of Medline, Embase, Web of Science, Scopus, and CINAHL was conducted per PRISMA 2020. Eligible studies were primary observational studies reporting objective vascular outcomes in night shift workers. Risk of bias was assessed using JBI critical appraisal tools. Narrative synthesis with vote-counting was used; meta-analysis was not feasible due to heterogeneity.

**Results:**

Ten studies from 2,257 records met the inclusion criteria across five vascular outcome domains. Cumulative night shift exposure was associated with increased arterial stiffness (PWV +1.29 m/s over 3 years) and reduced endothelial function (RHI −0.054). Associations were frequently attenuated to non-significance after covariate adjustment, and CIMT findings were directionally inconsistent. Poor sleep quality independently predicted higher CIMT in healthcare workers. Former shift workers showed persistent cerebrovascular white matter changes.

**Conclusion:**

Current evidence suggests possible associations between night shift work and arterial stiffness, though findings for other vascular outcomes remain inconsistent. No primary data exist for radiographers. The hypothesis of synergistic vascular injury from combined radiation and circadian disruption is biologically plausible but requires direct investigation through longitudinal, occupation-specific studies with standardized vascular outcome measures.

**Study Registration:**

The review was developed and recorded following the PRISMA guidelines and has been registered with PROSPERO (CRD420261378070) on 24 April 2026.

## 1. Introduction

The International Labour Organization defines night shift work as employment requiring at least three hours of work between 22:00 and 06:00 on a regular or rotational basis, and it accounts for 15–25% of the workforce in industrialized nations (Puttonen et al., 2010). With a projected 20.5 million deaths in 2021—roughly one-third of all deaths worldwide—cardiovascular disease (CVD) continues to be the major cause of death. ^1^ Shift work is one of the modifiable occupational risk factors that has drawn more epidemiological attention. Meta-analytic evidence indicates that shift workers have a 5% increased risk of ischemic stroke and a 23–40% higher relative risk of ischemic heart disease when compared to daytime workers. ^2^ ^,^ ^3^ Healthcare workers, who are crucial to providing services around the clock, are especially vulnerable to this exposure. According to new research, sleep deprivation, circadian rhythm disturbance, and neuroendocrine dysregulation—all of which lead to changes in arterial structure and function—may underlie the detrimental cardiovascular effects of working nights. ^4^

A distinct occupational category, radiographers are subjected to unpredictable shift patterns and long-term low-level ionizing radiation. This dual-exposure profile offers a medically sound foundation for developing hypotheses about compounded vascular risk. A mechanistic and epidemiological basis for this study is provided by indirect data from healthcare and occupational populations, even though no primary investigations have specifically looked at vascular function measures in radiographers. ^5^ ^,^ ^6^

Carotid intima-media thickness (CIMT), pulse wave velocity (PWV), augmentation index (AIx), reactive hyperaemia index (RHI), and cerebrovascular white matter integrity are examples of non-invasive vascular function parameters that offer reliable, sensitive indicators of subclinical CVD that occur years or decades before clinical events. ^7^ ^,^ ^8^ Early detection of vascular risk trajectories prior to the onset of irreversible clinical illness is made possible by their evaluation in occupational cohorts.

The objectives of this systematic review are to: (i) To determine whether night shift work, or its mechanistic proxy of sleep disturbance, is independently associated with subclinical vascular dysfunction and structural arterial changes in working-age adults employed in healthcare or shift-based industrial occupations; (ii) To evaluate the strength and consistency of evidence across objective vascular outcome domains and assess the applicability of findings to radiographers as a distinct occupational group; (iii) assess the extent to which this indirect evidence may be applicable to radiographers as a distinct occupational group, explicitly acknowledging the fundamental indirectness of all available evidence.

These objectives were operationalised through a structured research question, detailed in the methods section.

## 2. Methods

### 2.1. Protocol and registration

The Preferred Reporting Items for Systematic Reviews and Meta-Analyses (PRISMA) 2020 statement ^9^ was followed in the conduct and reporting of this systematic review. Supplementary Table 1 contains the PRISMA 2020 checklist. This review was prospectively registered with PROSPERO prior to the search (CRD420261378070), and the specified process was followed exactly.

As mechanistic proxies for circadian disturbance linked to night shift scheduling, sleep-related variables (sleep duration and quality) were incorporated as a pre-specified secondary exposure category. They are clearly marked as such throughout and were examined independently from direct shift-work exposure studies. Several publications from the same cohort were not considered distinct populations, but rather connected datasets reflecting consecutive follow-up analyses.

The following broad research question served as the basis for this systematic review: Among working-age adults employed in healthcare, healthcare-adjacent, or industrial settings, does exposure to night shift work or its mechanistic proxy of sleep disturbance — independently increase the risk of subclinical vascular dysfunction and structural arterial changes as measured by objective, instrument-based vascular parameters?

### 2.2. Eligibility criteria

Studies were eligible if they: (i) used a cross-sectional, cohort, or case-control observational design; (ii) enrolled adults (≥18 years) working in healthcare, healthcare-adjacent, or industrial settings with verifiable night shift exposure; (iii) defined night shift work as at least three hours of work between 22:00 and 06:00 on a regular or rotating basis; and (iv) reported at least one objective, instrument-measured vascular parameters, such as CIMT, PWV (any method), AIx, RHI, carotid plaque presence or total plaque area, or cerebrovascular imaging metric. Only English-language research papers were considered. Case reports, reviews, and editorials were not used as primary sources of evidence.

Since sleep disturbance is the most direct and quantifiable physiological effect of working nights, the pre-specified inclusion of sleep-proxy studies was warranted. Due to the fundamental difference in population type, one study ^10^ that examined sleep architecture in clinical ICA stenosis patients was only included as indirect mechanistic evidence; it was not included in the primary evidence synthesis and is not eligible for JBI appraisal.

### 2.3. Search strategy

An electronic search was carried out in PubMed/MEDLINE, Embase, Web of Science, Scopus, and CINAHL. For every database, the following Boolean search string was used and modified:

*(“night shift work” OR “shift work” OR “shiftwork” OR “rotating shift” OR “night work”) AND (“carotid intima-media thickness” OR “CIMT” OR “IMT” OR “arterial stiffness” OR “pulse wave velocity” OR “PWV” OR “augmentation index” OR “reactive hyperaemia” OR “endothelial function” OR “carotid plaque” OR “atherosclerosis” OR “white matter”)*

Filters applied: English language; humans. Reference lists of all included studies and relevant reviews were hand-searched.

**Figure 1.**
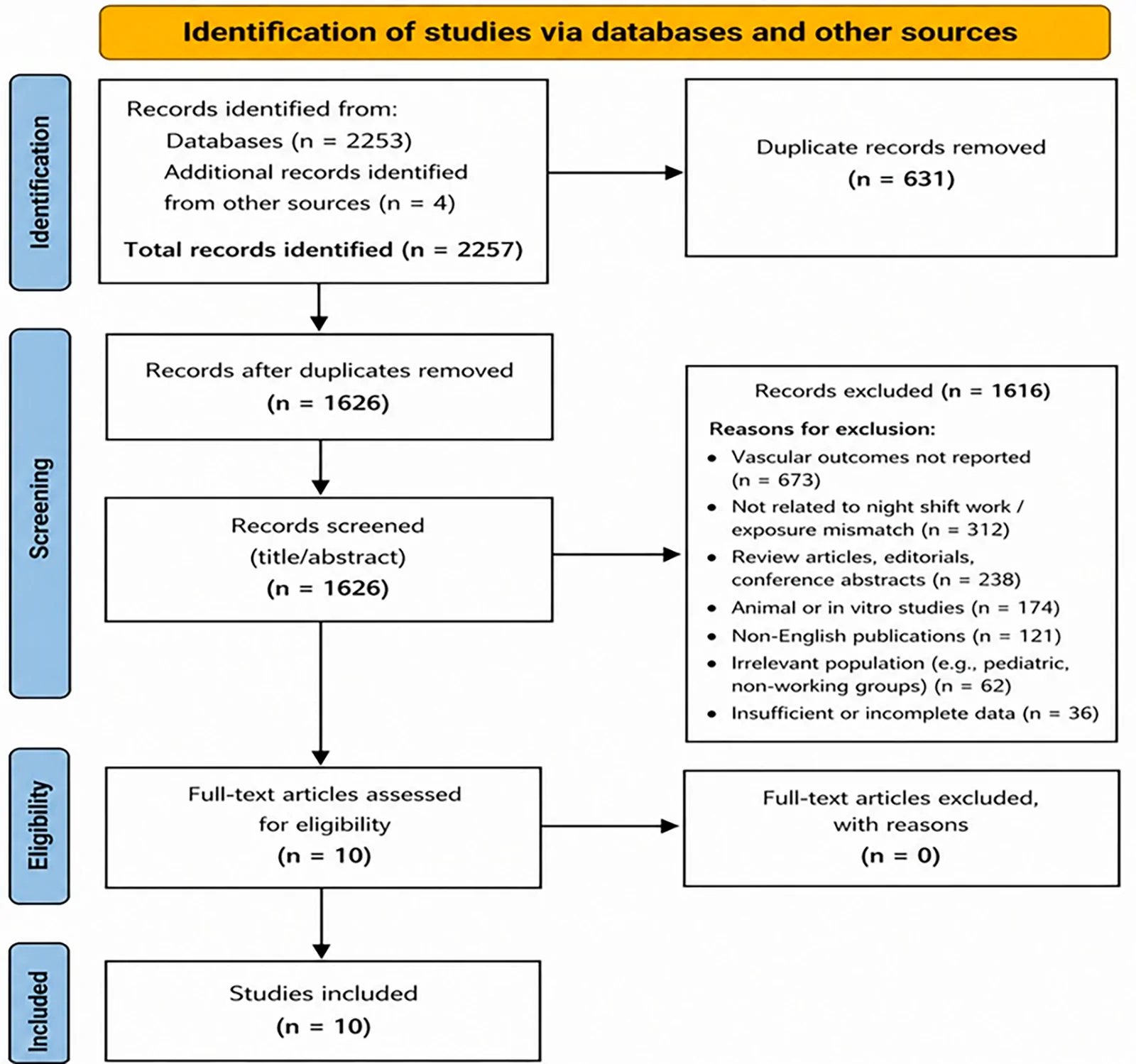
PRISMA flow chart.

A full-text evaluation of possibly eligible records was conducted after titles and abstracts were evaluated by two independent, blind reviewers. A third reviewer or consensus was used to settle disagreements. The research selection procedure is summarized in the PRISMA 2020 flow diagram ( Figure 1). First author and year, country, study design, population characteristics (occupation, n, age, sex), exposure definition and metric, vascular outcome(s) and measurement tool, adjusted effect estimate with 95% confidence interval (CI) when available, confounders adjusted for, and JBI appraisal score were all recorded on a standardized extraction form.

### 2.4. Risk of bias assessment

The JBI Critical Appraisal Checklist for Analytical Cross-Sectional research (8 criteria) and the JBI Critical Appraisal Checklist for Cohort Studies (11 criteria), if applicable, were used to evaluate the risk of bias in individual research. There were four possible ratings for each criterion: yes (Y), no (N), uncertain (U), and not applicable (NA). Studies were categorized as poor quality (<50%), moderate quality (50–74%), or high quality (≥75%). Independent appraisals were conducted by two reviewers; differences were settled by consensus.

In order to give a comparator quality measure (0–9 stars) that allowed for domain-level dissection across the Selection, Comparability, and Outcome/Exposure domains, the Newcastle-Ottawa Scale (NOS) was also used. Studies with a score of at least six were considered to be of excellent quality. Self-reported shift work exposure (information bias), healthy worker survivor bias (selection bias), cross-sectional design that precludes causal inference, and diverse CIMT assessment techniques were among the major concerns found throughout the evidence base.

## 3. Results

### 3.1. Selection of studies

Database searches (n = 2,253) and additional sources (n = 4) yielded a total of 2,257 records, as shown in Figure 1. 1,626 records remained after duplicates (n = 631) were eliminated, and they were filtered using the abstract and title.1,616 records were eliminated during the screening phase for the following reasons: lack of vascular outcomes (n = 673); unrelated to night shift work or exposure mismatch (n = 312); review articles, editorials, or conference abstracts (n = 238); animal or in vitro studies (n = 174); non-English publications (n = 121); irrelevant population (n = 62); and inadequate or incomplete data (n = 36). Ten full-text publications were then evaluated for eligibility; no additional exclusions were made.

Ten papers were ultimately included in the study as they satisfied all inclusion requirements.

Five vascular outcome domains were covered by the ten included studies: arterial stiffness/PWV (three studies; two from the same cohort), endothelial function/RHI (one study), CIMT (six studies; overlap with sleep proxy studies), carotid plaque/total plaque area (one study), and cerebrovascular white matter integrity (one study). Only the indirect mechanistic evidence from one other research ^10^ was kept. Table 1 summarizes the features of the study. Interestingly, two of the included studies ^11^ ^,^ ^12^ are not regarded as independent replications because they come from the same Norwegian industrial cohort.

**Table 1.** PECO framework.

Population (P)	Adults (≥18 years) employed in healthcare, healthcare-adjacent, or industrial settings with verifiable night shift exposure
Exposure (E)	Night shift work, defined as ≥3 hours of work between 22:00 and 06:00 on a regular or rotating basis; sleep disturbance (sleep duration and quality) was incorporated as a pre-specified secondary exposure category serving as a mechanistic proxy for circadian disruption
Comparator (C)	Day shift workers, non-shift workers, or reference groups not engaged in night shift work
Outcome (O)	Objective, instrument-measured vascular parameters, including carotid intima-media thickness (CIMT), pulse wave velocity (PWV, any method), augmentation index (AIx), reactive hyperemia index (RHI), carotid plaque presence or total plaque area, and cerebrovascular imaging metrics

**Table 2.** Characteristics of the studies included in the review.

Study design, Country	Population (n; occupation; age; sex)	Exposure matric	Vascular outcome(s)	Key findings
^13^Cross-sectional China	Chinese steelworkers n = 2,760 Mean age 43y; male	Current shift status; cumulative nights; duration; frequency	CIMT (B-mode duplex ultrasound; bilateral CCA; 7.5 MHz)	Mean CIMT 0.66 mm (SD 0.22) in shift workers; no significant association after full multivariable adjustment (β NR; p > 0.05)
^11^Prospective cohort (3 yr) Norway	Industrial workers n = 109 (84 SW; 25 DW) Mean age ~ 42y; mixed	Rotating shift (day/evening/night); years in shift work	cfPWV (SphygmoCor); CRP; sVCAM-1; sP-selectin	PWV Δ +1.29 m/s (SW) vs +0.11 m/s (DW) over 3 years; p < 0.05; CI NR
^12^[SAME COHORT as 2023a; 4-yr FU] Prospective cohort Norway	Same cohort as above (n = 84 SW; 25 DW) Extended follow-up	Rotating shift; night shift reduction intervention in one plant	PWV; central aortic BP; AIx; augmentation pressure	Night shift reduction associated with attenuation of PWV progression; AIx and AP non-significant; CI NR
^17^Cross-sectional (HCHS) Germany	Hamburg City Health Study n = 1,226 Mean age 62y; mixed	Current vs former vs never shift work	CIMT (CCA far-wall duplex); MRI PSMD (cerebrovascular WM)	Former SW: greater PSMD vs never-SW (effect size NR; p < 0.05); CIMT: NS after full adjustment
^19^Cross-sectional Hong Kong	Resident doctors n = 208; mean age ~ 27y Mixed sex	Sleep quality (PSQI score); on-call night duties [sleep proxy]	CIMT (carotid duplex ultrasound; CCA)	Higher PSQI (worse sleep) independently associated with greater CIMT; β NR; p < 0.05; CI NR
^15^— CARDIA Observational cohort USA	Community adults n = 617; age 37–52y 58% female; Black/White	Objective sleep duration (wrist actigraphy) [sleep proxy]	CIMT (mean of 20 measurements; CCA + bulb + ICA; ultrasound)	Each +1 h sleep: −0.026 mm CIMT in men (p = 0.02; 95% CI −0.047, −0.005); NS in women
^14^— NOMAS Cross-sectional USA	Community adults n = 1,553; mean age 64y Black/Hispanic majority	Self-reported nightly sleep duration; daytime sleepiness [sleep proxy]	Carotid plaque presence; total plaque area (TPA); CIMT (ultrasound)	Long sleep: OR 1.1/hour for plaque presence (95% CI NR; fully adjusted); short sleep: greater TPA
^18^Cross-sectional Germany	Employed general population n = 8,065 (of N = 15,010) Age 35–64y; mixed	Current night shift; cumulative nights/10 yr; lifetime total	Stiffness index (m/s; Endo-PAT); RHI (peripheral arterial tonometry); CIMT (duplex)	>660 shifts/10 yr: stiffness index +0.33 m/s (p < 0.05; CI NR); lifetime: RHI −0.054 (p < 0.05; CI NR); CIMT NS
^16^— SHIP Cross-sectional Germany	General population n = 2,437; wide age range Mixed sex	Self-reported total daily sleep duration [sleep proxy]	CIMT (CCA duplex ultrasound)	J-shaped association: minimum CIMT at 7–8 h; <6 h: +0.042 mm (age/sex adjusted; CI NR); >8 h: +0.084 mm
^10^[INDIRECT MECHANISTIC EVIDENCE ONLY — not primary evidence] Clinical observational; Russia	ICA stenosis/occlusion patients n = 24 (19 M; 5F) Clinical sample; mixed age	Degree of ICA stenosis/occlusion: <50%; 50–70%; occlusion	Polysomnography: sleep stage architecture (Stage II, SWS, REM)	ICA occlusion: SWS and REM disrupted (45% of cases); 50–70% stenosis: Stage II + SWS disruption; <50%: preserved

**Table 3.** Quality assessment of the studies.

Study	JBI Tool Applied	JBI Score	JBI Quality	NOS Selection (0–4★)	NOS Compariblity (2★)	NOS Outcome (0–3★)	NOS Total (0–9★)
^13^	Cross-sectional (8-item)	75%	High	★★★	★	★★	6
^11^	Cohort (11-item)	82%	High	★★★★	★★	★★	8
^12^ [same cohort as 2023a]	Cohort (11-item)	82%	High	★★★★	★★	★★	8
^17^	Cross-sectional (8-item)	75%	High	★★★	★★	★★	7
^19^	Cross-sectional (8-item)	75%	High	★★★	★	★★	6
^15^	Cohort (11-item)	88%	High	★★★	★★	★★	7
^14^	Cross-sectional (8-item)	75%	High	★★★	★	★★	6
^18^	Cross-sectional (8-item)	88%	High	★★★★	★★	★★	8
^16^	Cross-sectional (8-item)	75%	High	★★★	★	★★	6
^10^	Cross-sectional (8-item)	63%	Moderate	★★	★	★★	5

### 3.2. Study characteristics and risk of bias

Detailed study characteristics and risk of bias assessments are provided in Table 2 and Table 3. Overall, the included studies were of moderate-to-high methodological quality based on JBI and NOS assessments, although common limitations included cross-sectional design, self-reported exposure, and potential residual confounding.

The included studies demonstrated generally consistent relationships between night shift exposure, or its sleep-disturbance proxy, and adverse subclinical vascular indicators across five vascular outcome domains: arterial stiffness, endothelial function, carotid intima-media thickness, carotid plaque burden, and cerebrovascular white matter integrity. Although they are suggestive of potential cumulative vascular effects, the majority of data come from cross-sectional designs, which record group-level changes rather than longitudinal development and cannot demonstrate causal directionality.

Different studies employed different measurement instruments. Pulse Wave Velocity (PWV) and Augmentation Index (AIx) are complementary measures of arterial stiffness — PWV quantifies the speed of pressure wave propagation along the arterial wall, while AIx reflects the contribution of reflected waves to central aortic pressure; higher values of both indicate stiffer vessels. The Stiffness Index (SI), obtained from finger photoplethysmography, is an easier method for estimating large artery stiffness that can be used in large-scale population studies. The Reactive Hyperemia Index (RHI), obtained using peripheral arterial tonometry (EndoPAT), measures endothelial function in microvessels, while low values suggest reduced vasodilation. The carotid intima-media thickness (CIMT) measurement with B-mode ultrasonography is a well-known structural measure of atherosclerotic disease, although there is inconsistency across studies due to differences in anatomical localization, number of segments measured, and instrumentation. Total Plaque Area (TPA) takes this further by calculating the total cross-sectional area of all carotid plaques. Peak Skeletonized Mean Diffusivity (PSMD) is an advanced structural imaging parameter using diffusion tensor MRI.

The results on arterial stiffness were the most reliable. In the GHS study (n = 8,065), a dose-dependent effect was shown, where the stiffness index increased from 0.33 m/s after more than 660 night shifts (p < 0.05). On the longitudinal level, Skogstad et al. (2023a) reported an increase in PWV of 1.29 m/s in shift workers compared to 0.11 m/s in day workers over three years, with the follow-up studies showing that lowering shift work frequency slows down this process (Skogstad et al., 2023b – same sample size). The only result on RHI was observed in the GHS study, which showed worse endothelial function (−0.054 points for lifetime years of night work; p < 0.05).

CIMT findings were the most heterogeneous. Wang et al. ^13^ found elevated CIMT in shift workers that was attenuated after full adjustment, while sleep-proxy studies showed more consistent signals — worse subjective sleep quality independently predicted CIMT in resident doctors (Agudelo et al. ^14^), objective short sleep inversely correlated with CIMT in men (Sands et al. ^15^), and a J-shaped relationship emerged between sleep duration and CIMT in the SHIP cohort (Wolff et al. ^16^), with both short (<6 h) and long (>8 h) sleep associated with higher values. Agudelo et al. similarly identified a J-shaped pattern for TPA and plaque presence. Finally, Rimmele et al. ^17^ found greater PSMD in former shift workers after adjustment, with the persistence of this signal beyond exposure cessation suggesting that cerebrovascular changes may not fully reverse following shift-work discontinuation.

## 4. Discussion

This systematic review found that night shift work is consistently associated with adverse subclinical vascular changes across healthcare and occupational populations. The most reproducible finding was an association between cumulative night shift exposure and increased arterial stiffness. Skogstad, Aass, et al. ^11^ demonstrated a PWV increase of +1.29 m/s in shift workers versus +0.11 m/s in day workers over three years, and Skogstad, Goffeng, et al. ^12^ further showed that a night shift reduction intervention attenuated this PWV progression, strengthening the inference that the exposure-outcome relationship is modifiable. At the cross-sectional level, Jankowiak et al. ^18^ reported that exceeding 660 cumulative night shifts per decade was associated with a stiffness index increase of +0.33 m/s, and that lifetime night shift exposure was independently associated with reduced RHI, indicating endothelial dysfunction. These findings are biologically coherent with mechanisms involving chronic circadian misalignment, suppressed nocturnal melatonin, dysregulated autonomic tone, and elevated systemic inflammation.

CIMT associations were directionally inconsistent. Wang et al. ^13^ found no significant CIMT association after full multivariable adjustment in 2,760 steelworkers, and Rimmele et al. ^17^ similarly reported non-significant CIMT findings after adjustment, though former shift workers showed greater cerebrovascular white matter disruption. Sleep-proxy studies produced mixed CIMT signals: Sands et al. ^15^ found each additional hour of objective sleep associated with −0.026 mm CIMT in men only, Wolff et al. ^16^ identified a J-shaped relationship with minimum CIMT at seven to eight hours, and Sethi et al. ^19^ found worse sleep quality independently associated with greater CIMT in resident doctors, while Agudelo et al. ^14^ reported long sleep duration associated with greater carotid plaque presence. This heterogeneity likely reflects genuine biological complexity across distinct vascular territories and outcome timescales rather than the absence of effect.

No included study examined radiographers directly, and all implications for this population remain inferential. Radiographers plausibly share core mechanisms of circadian disruption and occupational stress with the industrial and healthcare populations studied. However, their unique exposures — chronic low-level ionising radiation, prolonged lead garment use, and acute emergency imaging demands — represent additional potential vascular stressors entirely absent from all included evidence. Both ionising radiation and night shift work independently activate NF-κB inflammatory pathways and promote vascular senescence, making a synergistic interaction biologically plausible, though wholly without empirical support. Primary investigation in radiographers is the necessary and most urgent next step.

## 5. Limitations

A number of factors must be considered when interpreting the review’s results. Eight of the 10 primary studies used cross-sectional designs at the study level, making it impossible to draw conclusions about causality. The two longitudinal investigations ^11^ ^,^ ^12^ are not independent replications since they come from the same cohort. Direct comparison was hampered by the varying operationalization of night shift employment among studies, which varied according to present status, cumulative shift count, years of exposure, or sleep length. Directional inconsistency was caused by significant variations in the CIMT methodology’s equipment, segment number, and site coverage. Because chronotype, nutrition, and obstructive sleep apnea were not taken into account in a number of studies, residual confounding is probable, and the healthy worker effect likely leads to a systematic underestimation of genuine vascular correlations.

Effect sizes cannot be explicitly pooled at the review level due to the lack of meta-analysis. Funnel plot evaluation was not possible since there were fewer than 10 studies for each outcome domain, and positive publication bias may have inflated reported relationships. Additional selection bias is introduced by the restriction to English-language publications and the lack of a systematic search for grey literature. Careful subclassification was necessary because of the conceptual heterogeneity of the included research, which comprised one clinical ICA stenosis cohort, sleep-proxy studies, and direct shift-work exposure. ^10^ was only included as an indirect mechanistic background, omitted from the primary synthesis and JBI evaluation, and sleep-proxy studies were handled as a predetermined secondary exposure category. The most significant deficit for future-focused research is that all primary information comes from industrial and general working populations, and no included study specifically looked at radiographers. This significantly restricts occupational transferability.

## 6. Conclusions

According to available data, night shift employment is frequently associated with negative vascular indicators in both healthcare and occupational groups. These results should be viewed with caution since they are mostly observational and represent variations between groups rather than longitudinal advancement. Although there is no main research for this demographic, it is plausible that radiographers are at higher vascular risk based on indirect evidence. Therefore, any implications for radiographers are openly hypothesis-driven and should not be taken as evidence-based. To establish this association, direct, carefully planned prospective trials are needed. Future studies should prioritize radiographers directly and concentrate on longitudinal designs, standardized vascular outcome protocols, pre-registered techniques, and occupation-specific cohorts.

## Data Availability

Figshare: Dataset for “Night Shift Work and Vascular Function Parameters: A Systematic Review of Occupational and Healthcare Evidence with Implications for Radiographers” https://doi.org/10.6084/m9.figshare.32397771. ^21^ This project contains the following underlying data:
•
Table 1: PECO Framework. •
Table 2: Characteristics of the studies included in the review. •
Table 3: Quality Assessment of the studies. • Figure 1: PRISMA flow chart. All data have been fully de-identified in an accordance with the safe Harbor method, following HIPPA guidelines ( https://www.hhs.gov/hipaa/for-professionals/special-topics/de-identification/index.html#standard). ^20^ Data are available under the term of the creative common attribution 4.0 International license (CC-BY 4.0). Figshare: The PRISMA flowchart for Night Shift Work and Vascular Function Parameters: A Systematic Review of Occupational and Healthcare Evidence with Implications for Radiographers https://doi.org/10.6084/m9.figshare.32397771. ^21^
