# Supplementary material for: Night Shift Work and Vascular Function Parameters: A Systematic Review of Occupational and Healthcare Evidence with Implications for Radiographers
Source: F1000Res. 2026 Jun 30;15:1032. [Version 1] doi: 10.12688/f1000research.182990.1 (PMC13579024; doi:10.12688/f1000research.182990.1)
Supplement: Peer review history

# Reviewer report: Reviewer response for version 1

**DOI:** 10.5256/f1000research.201984.r519259

**Published:** 2026-09-15

**Authors:** Pratik Phansopkar

**Affiliations:** 1 Musculoskeletal Physiotherapy, School of Physiotherapy, Bharati Vidyapeeth (Deemed to be University) Medical College and Hospital Sangli, Sangli, Maharashtra, India

Every occupation bears certain consequences when practiced over a period of duration for consequetive years.

 Night Shift mode of work, in every aspect impacts the physiological well-being of the employee. Radiographers population with exposure to radiations may further have impact on vascular system. Hence the Systematic Review conducted by the authors is important and provides a strong association between night shift work mode and arterial stiffness.

 The objectives of this Systematic Review are specifically structured, and the methodology is well designed and appropriate, and have utilized appropriate statistical analysis.

Are the rationale for, and objectives of, the Systematic Review clearly stated?

Yes

Is the statistical analysis and its interpretation appropriate?

Yes

If this is a Living Systematic Review, is the ‘living’ method appropriate and is the search schedule clearly defined and justified? (‘Living Systematic Review’ or a variation of this term should be included in the title.)

Not applicable

Are sufficient details of the methods and analysis provided to allow replication by others?

Yes

Are the conclusions drawn adequately supported by the results presented in the review?

Yes

Reviewer Expertise:

Musculoskeletal, Orthopaedic, Manual Therapy.

I confirm that I have read this submission and believe that I have an appropriate level of expertise to confirm that it is of an acceptable scientific standard.

# Reviewer report: Reviewer response for version 1

**DOI:** 10.5256/f1000research.201984.r501334

**Published:** 2026-08-08

**Authors:** Kumaraguruparan Gopal

**Affiliations:** 1 Gulf Medical University, Ajman, Ajman, United Arab Emirates

This systematic review has clear research objectives, well organized with an important occupational health topic and a comprehensive literature search. The manuscript highlighted the significant research gap regarding vascular function in radiographers those who working night shifts.

 The conclution should be more consisteance with the available research evidence. Most of the studies included in this research is cross sectional. So, the authors can avoid imlyication of the radiographers are at vascular risk without direct evidence.

Are the rationale for, and objectives of, the Systematic Review clearly stated?

Yes

Is the statistical analysis and its interpretation appropriate?

Yes

If this is a Living Systematic Review, is the ‘living’ method appropriate and is the search schedule clearly defined and justified? (‘Living Systematic Review’ or a variation of this term should be included in the title.)

Yes

Are sufficient details of the methods and analysis provided to allow replication by others?

Yes

Are the conclusions drawn adequately supported by the results presented in the review?

Partly

Reviewer Expertise:

Cardiorespiratory Physiotherapy

I confirm that I have read this submission and believe that I have an appropriate level of expertise to confirm that it is of an acceptable scientific standard.
